# Comparison of Open and Closed Nailing for Femoral Shaft Fractures: A Retrospective Analysis

**DOI:** 10.7759/cureus.16030

**Published:** 2021-06-29

**Authors:** Muhammad Tahir, Nadeem Ahmed, Ahmad Faraz, Hassan Shafiq, Mohammad Noah Khan

**Affiliations:** 1 Orthopaedics, Jinnah Postgraduate Medical Center, Karachi, PAK; 2 Orthopaedics and Traumatology, Jinnah Postgraduate Medical Centre, Karachi, PAK; 3 Trauma and Orthopaedics, Leeds Teaching Hospitals NHS Trust, Leeds, GBR; 4 Trauma and Orthopaedics, Royal National Orthopaedic Hospital, London, GBR; 5 Trauma and Orthopaedics, Royal Victoria Hospital, Belfast, GBR

**Keywords:** femoral nail, open reduction, closed reduction, restrospective analysis, comparison

## Abstract

Introduction

Open and closed nailing are the two reduction methods used for the fixation of femoral shaft fractures. The study aims to assess the clinical and functional outcomes of open and closed nailing for closed femoral shaft fractures.

Methodology

A total of 398 patients who underwent intramedullary nailing fixation of nonpathological femoral shaft fracture between January 2016 to December 2019 were reviewed retrospectively. Two hundred seventy-four underwent closed nailing, and 124 were considered for open nailing.

Results

The primary outcome reviewed was the union rate of fracture. Other outcomes analyzed were complications, intraoperative blood loss, time to union, and the duration of the procedure. Patients in the open group had a union of fracture in 15.71 weeks, closed nailing group had a union in 15.53 weeks (p-value 0.495). Patients with open nailing had a mean Radiological union scale in tibial (RUST) fracture score of 11.435, whereas the closed nailing group had a mean of 11.664 (p-value 0.187). Operative time was higher in the open group when compared to the closed nailing group (p-value 0.000). However, intraoperative blood loss was more in open nailing in comparison to closed nailing. Furthermore, 15 patients with closed nailing had non-union, whereas 11 had non-union after open nailing (p-0.204). Superficial infection and deep infection requiring debridement were equally observed among the two treatment groups.

Conclusion

Fixation of femoral shaft fractures with open nailing has similar outcomes in union rates, time to union, and rates of significant complication similar to those of close nailing.

## Introduction

Femoral shaft fractures are usually due to high energy trauma, such as road traffic accidents. The recent epidemiological study reports that 10 to 21 such fractures are around 100,000 person-years [1,2]. Road traffic collisions and fall from height together contribute to 40.5% of all injuries, and again, femoral fractures are commonly observed within this mechanism of injuries [3]. Intramedullary nailing (IMN) is one method to reduce femoral shaft fractures, with good clinical and functional outcomes [4]. Fakhry et al .observed that early surgical stabilization is associated with curtailment in complications and mortality [5]. An intramedullary nail is a metal rod inserted into the medullary cavity of bone and over the fracture site to support the fractured bone [6]. Advantages include early functional use of the limb, reduced hospital stay, an early union of the fractured bone [7].

Closed nailing techniques have been well defined with IMN of the femur [8]. In contrast, if they alone prove inadequate for achieving desire reduction, the open nailing fixation methods are recommended. Complications such as the risk of infected metalwork, non-union secondary to disruption of fracture hematoma, wound and deep tissue infections have been reported due to open IMN of close shaft of femur fracture [9]. Open and close nailing methods are the two preferred methods used by surgeons, depending on co-morbid, availability of operative room equipment such as C-arm and fracture table, fracture pattern and associated injuries (i.e., spinal injury, floating knee injury, concomitant acetabular fracture. Healing time taken by the fracture fixed with open intramedullary nailing is higher than closed nailing [10]. Schell et al. found that earlier healing time with close nailing is due to non-disturbance of fracture haematoma and reduced tissue damage [11].

We conducted this study to investigate the open and closed methods during the reduction of a femoral fracture. We hypothesize that there is no difference between these two methods regarding our primary outcome of time to fracture union or secondary outcomes of the duration of surgery, intraoperative blood loss and RUST (radiological union scale in tibial fractures) score. We used the RUST score as a surrogate measure of radiographic union, although we are aware that it is validated for tibia fractures primarily.

## Materials and methods

This is a retrospective study on patients treated at a major trauma center according to the Helsinki declaration with informed patient consent. IRB waiver was received. The present study was conducted between January 2016 to December 2019, where we reviewed medical records of all patients with closed femoral shaft fractures.

Inclusion Criteria

Patients with acute traumatic fractures or fractures of the femoral shaft among individuals older than 17, who were subsequently managed with open or closed intramedullary nailing, were included in our study. Based on previously published trials, a minimum follow-up of 6 months was required.

Exclusion Criteria

Patients younger than 17 or those with open fractures, pathological fractures, atypical pathological fractures using bisphosphonate, patients who refused to participate in the study and patients with less than six months of radiological follow-up were excluded from the study. 

Patient demographic data included age and gender, can be reviewed in Table 1. Mechanism of injury, smoking status, body mass index (BMI) and American Society of Anaesthesiology (ASA) are also shown in Table 1. Patients underwent routine radiographic follow-up at two weeks, six weeks, three months, and six months postoperatively. After six months of follow-up, patients were evaluated at either eight months or nine months postopera­tively, depending on surgeon preference, and at 12 months postoperatively. Patients were further evalu­ated at either 18 months or 24 months postoperatively. After 24 months, pa­tients were followed up in case of a clinical complaint or on a required basis. Radiographic evaluation was made after retrospectively reviewing bridging callus on 3 to 4 cortices seen on anteroposterior and lateral radiographs at the time of union. We did include the radiological union scan of Tibia (RUST) to assess the healing of tibial fractures following intramedullary nailing. Patients were not allowed to use external bone stimulators to help them with fracture healing. We also analyzed factors such as length of hospital stay, length of follow-up, an infection that require a return to the operating room, revision of fixation requiring the return to the operating room, and non-union or delayed union requiring the return to the operating room, time is taken for the radiographic union. Patients were further stratified as an open or closed group based on fracture pattern, anatomic deformity. If the fracture wasn't reduced with closed nailing, then it was converted to open nailing. Closed nailing was defined as reduction achieved through a smaller incision (<1cm), with an instrument such as bone hooks or ball-spike pushers to assist the indirect reduction of fracture fragments. Open nailing was defined as reduction with incision (>1cm long) to facilitate direct reduction of fracture fragments, where plates, cables and plates were used to provide temporary stabilization.

Three hundred ninety-eight patients underwent intramedullary nailing, 274 patients underwent closed nailing, whereas 124 were considered for open nailing as per the criteria defined above. We used analy­sis of variance for continuous variables, and chi-square was used for categorical variables. Statistical analy­sis was performed using SPSS 20 statis­tics software (IBM Corp, Armonk, New York).

## Results

Demographic data can be reviewed in Table 1, there was 186 male and 80 females in the closed nailing group, whereas open nailing had 80 males and 44 females. The overall mean age of the patients included was 35.93 years (range, 17-87 years). The mean age in the closed reduction group was 39 years, and the mean age in the open reduction group was 32.68 years. Road traffic accident and fall were the most common cause of injury identified in the two treatment groups (Table I). Patients in open reduction group who had union did so in a mean of 15.53 weeks vs a mean of 15.71 in the closed nailing group (P=0.495).RUST score was found equal between the two treatment group with a p-value of 0.187. The mean Intraoperative blood loss in the open nailing group was recorded to be 150.06, i.e. much higher, 38.77 in the close nailing group. All these surgical outcomes can be seen in Table 2.

**Table 1:** Demographic data Demographic data showing Open vs Closed Nail comparison, also highlighting co-morbids & American Society of Anesthesiologists (ASA) Grade

	Closed Nailing (274)	Open Nailing (124)	p-value
Age in years	39.18 ± 13.892	32.68 ± 5.274	0.000
Gender			
Male	186	80	0.509
Female	88	44	
Mechanism of Injury			0.001
Road Traffic Accident	150	86	
Fall	114	29	
Assault	10	9	
Smoker	45	20	0.941
Body Mass Index (kg/m^2^)	28.9503±3.93815	27.916±4.04786	0.017
American Society of Anesthesiologists Grade			0.844
I	73	32	
II	93	44	
III	62	24	
IV	46	24	

**Table 2:** Surgical Outcomes Surgical outcomes of open vs closed Nailing Femoral Fractures, which includes times taken by fracture to undergo union, RUST(radiographic union score for tibial (RUST), average procedure time, intraoperative blood loss

	Closed Nailing (274)	Open Nailing (124)	p-value
Time to Fracture Union in weeks	15.53 ± 2.481	15.71 ± 2.505	0.495
RUST Score	11.664 ± 1.44890	11.4355 ± 1.89240	0.187
Duration of Surgery in minutes	90.11 ± 14.538	107.94 ± 6.718	0.000
Intraoperative Blood loss in millilitres	38.77 ± 1.127	150.061 ± 1.456	0.000

Complications

Twenty-two patients had complications with close nailing, whereas 12 patients had complications who underwent open nailing. However, the p-value was recorded to be 0.112. Fifteen patients in the closed group had a non-union treated with closed nailing, whereas 11 patients in the open group had non-union. This difference was although not found to be statistically significant. Seven patients were required to return to the op­erating room to treat deep infec­tion, four patients (one in the closed reduction group and three patients in the open nailing (p-value 0.500). Only three patients were found to have a superficial infection after close nailing. Comparison of complications can be reviewed in table 3 as shown below

**Table 3:** Complications Post-op Complications Open vs Closed Nailing femoral fractures

	Closed Nailing (274)	Open Nailing (124)	p-value
Total No. of complications	22	12	0.112
Non-union	15	11	0.204
Superficial Infection	3	0	0.242
Deep infection requiring debridement	4	3	0.500

## Discussion

Several studies have been conducted to evaluate the outcomes of open vs closed intramedullary nailing for the treatment of femoral shaft fractures [12,13]. The results of this study seem to corroborate the findings published by Telgheder [14]. He found an overall rate of 91.6%, but there was no significant difference in the union rates between either the open or closed methods of femoral fractures. We observed a similar pattern in our study, where 94.5% of patients were recorded to have union after close nailing and 91.6% patients after open nailing had a union in their fracture; however, there was no statistical significance observed (p-value 0.495). Telgedher further found the meantime to union was 5.6 months, slightly longer than that seen in our study (3.7 months) [14].

Harper published a case series on closed and open intramedullary nail­ing, where he reported an equal rate of union among two treatment groups, with the equal time taken to achieve union. He further found a higher rate of malunion with closed intramedullary nailing [15]. Our findings are similar to a study published by Harper, where an almost equal number of patients had malunion among two groups. The time required to achieve union was nearly similar. However, Tahririan et al. published a study to compare open and close intramedullary nailing, where he reported one patient with malunion after open intramedullary nailing [16]. However, the meantime to attain union was 3.5 months. These findings are in contrast to our study. Telgheder et al. found that around 8.6% of patients required revision surgery, in the closed group, compared to 16.2% in the open group. They did not find any statistically significant difference between both these groups [14]. The vast majority of revisions (9/13 patients) were to treat subsequent fracture non-union within these patients. However, none of our patients underwent a re-do surgery.

Rascher et al. found that 42 femoral fractures fixed with closed intramedullary fixation restored normal anatomy altogether [17]. The literature review shows several studies on the closed method, but there are very few comparative studies between open and closed methods. The advantages of closed nailing over open nailing operation are still a debatable topic-Rokkanen et al. report slightly better results for closed nailing over open nailing [18]. However, Leighton conducted a two-year follow-up study to compare open and closed nailing. He found that 92% of patients with closed technique and 97% with open nailing had no significant difference in clinical outcomes [19]. He concluded that when a closed technique is attempted, preparations should also be made to open the fracture to achieve an adequate degree of reduction if closed reduction is unsuccessful. Gourishankar et al. [20] found shorter time to radiographic union and lower infection rates in patients with femoral shaft fractures treated with close reduction compared with those managed with open reduction.

Open nailing represents a useful technique for types of fractures that are not reduced using closed methods. These patients include those with co-morbidities multiple associated traumatic injuries and obese patients in whom external manipulation is a challenge. However, the fact that the procedure lasts longer causing complications and an excessive amount of radiations. King et al. published a review of 112 cases who underwent closed nailing, he found that four patients develop an infection; 7% of cases had shortening of the limb between 1 and 2cm [21]. The present study reports that seven patients had infections that underwent closed techniques, with four patients having deep infection requiring washout. We found that the closed nailing technique is quick with the meantime significantly better than the open technique. However, the use of x-rays during closed technique surgery is more when compared to the open technique. Chou et al. [22]. Reported that cancer incidence is observed to be higher in orthopaedic surgery procedures due to overly use of mobile intensifier images.

Salawu et al. [23] studied the clinical outcomes of closed femoral shaft fractures after open intramedullary nailing. The time to radiological fracture union was 14.0 ± 1.2 weeks, and two patients had malunion, broken nails (4.7%), infection, loosening of the distal screw, and limb length discrepancy (2.3% each). The present study found complications among 12 patients, with three superficial infections and 11 with malunion. This discrepancy in results could be due to our higher sample size.

Telgheder et al. found no difference among open vs closed intramedullary nailing in terms of mean hospital stay. In contrast, operative time was longer in the open reduction group than in the closed group [15]. The current study observed a similar pattern in terms of operative timings as reported by Telgedher, we found closed nailing was comparatively quicker than open nailing ( p=0.000).

A detailed review by Harper et al. in 1985 found no significant difference in hospitalization times between the open or closed reduction methods groups [15]. They found higher operative morbidity in patients undergoing an open reduction, but closed reductions seemed to result in higher intraoperative complications. Closed reduction methods also had a higher rate of malrotation, although the incidence of postoperative complications was similar in both groups [6].

The concern regarding a higher infection rate with an open fracture due to a larger wound and increased blood loss has also not been seen in our study thus far. We have found that though the difference in infection rates between the two groups is negligible, the closed group had a higher rate of superficial and deep infections when compared to the open group alone. We overlooked a consistent reason for this discrepancy between the two groups in our study. We also found that within the open reduction group. However, the operative time was longer, with a more significant degree of blood loss. Malik et al. studied open and closed nailing method, where he analysed the relationships between deep infection and non-union and the pre-and peri-operative factors of age, ASA score, indication for nailing, the use of reaming, the use of antibiotics.. We found a similar pattern in our study. There was no significant association between type of procedure and ASA score (p-value 0.844). There was no significant difference in the parameters such as smoking (p=0.941) and body mass index (p-0.17).

Limitations and Strength

We accept that there are limitations to the study that we have carried out, primarily regarding selection bias, as this is a retrospectively designed study, with an unequal number of patients in each group. We accept that this may skew the results as we may not have had enough patient is in each group to achieve a statistically significant difference between our groups. We have also used the RUST score, which was primarily validated for use in tibial fractures to quantify the degree of radiographic union, and felt this is the most relevant tool available to us as there are no comparative scores that have been validated exclusively for use within femoral fractures. We also accept that though we have attempted to quantify union radiographically, we have not done a functional outcomes evaluation of the patients and that radiographic union may not necessarily correlate with clinical satisfaction amongst our cohort. 

However, we feel that our study of 398 patients in total represents the largest review available in the literature to compare both open and closed methods of femoral fracture and their subsequent outcomes. We have also further strengthened the current literature evidence that reducing femoral fractures does not significantly impact the union rates or time to the union within these injuries. However, the quality of reduction is key to prevent further sequelae of non-union or mal-union in these subsets of patients.

## Conclusions

It is our view that though closed nailing of femoral fracture is still the first line of treatment, should there be any doubt or difficulty in achieving satisfactory reduction, then open nailing should not be hesitated to achieve the optimum outcome of fracture union, with little associated risk or side-effects of the open procedure, as we have demonstrated in our study.
